# EBP50 inhibits EGF-induced breast cancer cell proliferation by blocking EGFR phosphorylation

**DOI:** 10.1007/s00726-012-1277-z

**Published:** 2012-04-04

**Authors:** Wenfang Yao, Duiping Feng, Weihua Bian, Longyan Yang, Yang Li, Zhiyu Yang, Ying Xiong, Junfang Zheng, Renyou Zhai, Junqi He

**Affiliations:** 1 Department of Biochemistry and Molecular Biology, Capital Medical University, Beijing, 100069 People’s Republic of China; 2 Department of Radiology, Beijing Chao-Yang Hospital, Capital Medical University, Beijing, 100020 People’s Republic of China

**Keywords:** EBP50, EGFR, Protein interaction, Phosphorylation, ERK1/2, PDZ, Breast cancer

## Abstract

Ezrin-radixin-moesin-binding phosphoprotein-50 (EBP50) suppresses breast cancer cell proliferation, potentially through its regulatory effect on epidermal growth factor receptor (EGFR) signaling, although the mechanism by which this occurs remains unknown. Thus in our studies, we aimed to determine the effect of EBP50 expression on EGF-induced cell proliferation and activation of EGFR signaling in the breast cancer cell lines, MDA-MB-231 and MCF-7. In MDA-MB-231 cells, which express low levels of EBP50, EBP50 overexpression inhibited EGF-induced cell proliferation, ERK1/2 and AKT phosphorylation. In MCF-7 cells, which express high levels of EBP50, EBP50 knockdown promoted EGF-induced cell proliferation, ERK1/2 and AKT phosphorylation. Knockdown of EBP50 in EBP50-overexpressed MDA-MB-231 cells abrogated the inhibitory effect of EBP50 on EGF-stimulated ERK1/2 phosphorylation and restoration of EBP50 expression in EBP50-knockdown MCF-7 cells rescued the inhibition of EBP50 on EGF-stimulated ERK1/2 phosphorylation, further confirming that the activation of EGF-induced downstream molecules could be specifically inhibited by EBP50 expression. Since EGFR signaling was triggered by EGF ligands via EGFR phosphorylation, we further detected the phosphorylation status of EGFR in the presence or absence of EBP50 expression. Overexpression of EBP50 in MDA-MB-231 cells inhibited EGF-stimulated EGFR phosphorylation, whereas knockdown of EBP50 in MCF-7 cells enhanced EGF-stimulated EGFR phosphorylation. Meanwhile, total expression levels of EGFR were unaffected during EGF stimulation. Taken together, our data shows that EBP50 can suppress EGF-induced proliferation of breast cancer cells by inhibiting EGFR phosphorylation and blocking EGFR downstream signaling in breast cancer cells. These results provide further insight into the molecular mechanism by which EBP50 regulates the development and progression of breast cancer.

## Introduction

Epidermal growth factor receptor (EGFR) is a member of the ErbB family of receptor tyrosine kinases. Overactivation of EGFR gives rise to the deregulation of this EGFR-dependent signaling network, which is involved in the development and malignancy of numerous types of human cancers including breast, head and neck, lung, bladder, colon, prostate, kidney, ovary, brain, and pancreatic cancers (Kolibaba and Druker 1997). The EGF receptor is the prototypical receptor with intrinsic protein tyrosine kinase activity (Klapper et al. 2000; Olayioye et al. 2000; Salomon et al. 1995). Upon ligand binding by its extracellular domain, EGFR forms homo- or hetero-dimers with the closely related ErbB receptors (such as ErbB2/Her2), and begins tyrosine autophosphorylation of its cytoplasmic domain (Klapper et al. 2000; Olayioye et al. 2000; Salomon et al. 1995). This autophosphorylation of the intracellular domain of EGFR is critical for signal transduction, since it creates docking sites for various signaling molecules such as molecules containing Src homology 2 or phosphotyrosine-binding domains. These interactions with downstream molecules create signaling complexes that lead to oncogenic responses by linking EGFR activation to numerous cytoplasmic signaling pathways (Klapper et al. 2000; Olayioye et al. 2000; Salomon et al. 1995). Therefore, identifying molecules that interact with the cytoplasmic domain of EGFR will contribute, not only to the elucidation of the regulatory mechanism of EGFR in cancer progression, but also to the development of new treatments for the uncontrolled growth of human cancers.

Many proteins have been reported to interact with the cytoplasmic domain of EGFR (Fedor-Chaiken et al. 2003; Kim et al. 2003). One recently reported protein was Postsynaptic density-95/Discs Large/ZO-1 (PDZ) Ezrin-radixin-moesin-binding phosphoprotein-50 (protein EBP50), also known as NHERF, NHERF1 (Lazar et al. 2004). EBP50 can contribute to biliary epithelial cell proliferation (Fouassier et al. 2009) and to the development of liver cancer (Shibata et al. 2003). However, EBP50 is also reported to suppress EGFR activity, which inhibits epithelial-to-mesenchymal transition (EMT) phenotypic changes in biliary cancer cells (Claperon et al. 2011). Results from Pan et al. (2006) and our previous study demonstrated that EBP50 can suppress breast cancer cell proliferation (Zheng et al. 2010b); but, its regulatory effects on EGFR signaling in breast cancer cells remains unknown. Therefore, the goal of this study was to further elucidate the role of EBP50 on EGF-induced breast cancer cell proliferation and EGFR signaling in breast cancer cells. Our results showed that EBP50 can inhibit EGF-stimulated breast cancer cell proliferation and EGFR pathway activation, supporting a role for EBP50 in inhibiting EGF-induced cell proliferation by blocking EGFR phosphorylation in breast cancer cells.

## Materials and methods

### Plasmids

The pBK-CMV-HA empty vector and pBK-CMV-HA-EBP50 expression plasmid were kindly provided by Dr. Randy Hall from Emory University. pSuper.puro EBP50 RNAi and pSuper.puro luciferase control RNAi plasmids were kindly provided by Margaret J. Wheelock from University of Nebraska Medical Center.

### Cell lines, cell culture and cell treatments

The human breast cancer cell lines MDA-MB-231 and MCF-7 were obtained from the American Type Culture Collection (ATCC, Manassas, VA). MDA-MB-231 cell was cultured in GIBCO RPMI 1640 medium (Hyclone, Logan, UT) and MCF-7 cell was cultured in Dulbecco’s modified Eagle’s medium (DMEM) (Forni et al. 2009) at 37 °C and 5 % CO_2_. Both media were supplemented with 10 % fetal bovine serum (FBS, Hyclone) and 1 % antibiotic–antimycotic agent (Life Technologies Inc, Grand Island, NY). Cells were grown to 80 % confluency for use.

MDA-MB-231 and MCF-7 cells were serum starved overnight, then treated with 100 ng/ml EGF (Sigma-Aldrich Chemical Corp., St. Louis, MO) for different time at 37 °C to detect the effect of EBP50 expression on EGFR-mediated signal transduction pathways.

### Stable transfection

For EBP50 stable overexpression, MDA-MB-231 cells were transfected with pBK-CMV-HA-EBP50 plasmid or pBK-CMV-HA vector, respectively, using FuGENE6 (Roche, IN) following the protocol reported previously (Konno et al. 2009). For EBP50 stable knockdown, MCF-7 cells were transfected with pSuper.puro EBP50 RNAi or control pSuper.puro luciferase RNAi plasmid, respectively, using Hifectin II (Applygen Technologies Inc, Beijing, China) following the protocol. 2 days following transfection, cells were transferred to 100-mm plates and cultured in selection medium with 350 μg/ml G418 (Amresco) for EBP50 overexpression and 0.5 μg/ml puromycin (Sigma-Aldrich) for EBP50 knockdown (Hu et al. 2008; Theisen et al. 2007). The medium was changed every 2 days to remove floating dead cells, and resistant colonies formed were harvested and plated in 24-well plates. Cell cultures were expanded and cultured for at least a month, and fractions were used for analysis of EBP50 expression by western blotting, with GAPDH expression as a protein loading control. Stably transfected cell pools were maintained and passaged in culture medium with G418 (200 μg/ml) or puromycin (0.25 μg/ml).

### RNAi-mediated transient EBP50 knockdown

Small interfering RNA (siRNA) duplexes directed against EBP50 (nucleotides to: 5′-GUCGACCACCAGCAGGCGCACGGCGUUG-3′) were synthesized by Sigma-Aldrich. To perform the rescue experiments of knocking down EBP50 in EBP50-overexpressed MDA-MB-231 cells (EBP-231 cells), the EBP-231 cells were grown to 80 % confluency in 35-mm dishes, transfected with 2 μl Lipofectamine 2000 (Invitrogen, Carlsbad, CA), and mixed with 36 pmol of the synthetic EBP50 siRNA. The cells were then serum starved overnight, stimulated, harvested and analyzed after 48 h of transfection.

### Cell proliferation assay

The Cell Counting Kit-8 (CCK-8, Dojindo, Kumamoto, Japan) colorimetric assay was conducted to measure the relative number of viable cells (Takeuchi et al. 2003). MDA-MB-231 and MCF-7 cells (3 × 10^3^ cells/well) were serum starved overnight, then treated with 100-ng/ml EGF for continuous stimulation and viable cells were detected at different days, respectively.

Thymidine analog 5-bromo-2’-deoxyuridine (BrdU, Cell Signaling Technology, Beverly, MA) incorporation assay was used to measure the DNA synthesis rate in proliferating cells as described previously (Wang et al. 2004). Cells (5 × 10^3^ cells/well) were spread onto 96-well plates and incubated for different days in cell culture media in the presence of serum combined with 100-ng/ml EGF stimulation to detect.

### Western blotting

Western blotting was performed as described before (Zheng et al. 2010a). In brief, sample aliquots corresponding to 25 μg of protein were resolved using 10 % sodium dodecyl sulfate-polyacrylamide gel electrophoresis (SDS-PAGE) for 1 h at 150 V and then transferred to nitrocellulose membrane. The blots were blocked in the blot buffer (2 % nonfat dry milk, 0.1 % Tween 20, 50 mM NaCl, 10 mM Hepes, pH 7.4) for at least 30 min and then incubated with primary antibody in the blot buffer for 1 h at room temperature or overnight at 4 °C. The blots were then washed three times with 10 ml of the blot buffer each and incubated for 30 min at room temperature with a horseradish peroxidase-conjugated secondary antibody in the blot buffer. Finally, the blots were washed three more times with 10 ml of the blot buffer each and visualized by enzyme-linked chemiluminescence (Amersham Biosciences, NJ). The phospho-EGFR, phospho-extracellular signal-regulated kinase 1/2 (ERK1/2) and phospho-AKT assays were preformed as previously described (Zheng et al. 2010a). Blots were quantified using the US National Institutes of Health Image 1.62 program. Protein levels were normalized with GAPDH, and the levels of phospho-EGFR, phospho-ERK1/2 and phospho-AKT immunoreactivity were normalized to the total EGFR, ERK1/2 and AKT immunoreactivity, respectively. The primary antibody specific for the EBP50 was purchased from BD Biosciences (San Jose, CA). Other primary antibodies specific for GAPDH, EGFR, phospho-EGFR (Tyr1173), phospho-ERK1/2 (Thr202/Tyr204), phospho-AKT (S473), total ERK1/2 and AKT were all bought from Cell Signaling Technology. Horseradish peroxidase-conjugated anti-mouse IgG and anti-rabbit IgG secondary antibodies were purchased from Amersham Biosciences.

### Statistics

All experiments were repeated at least three times. Results were analyzed using SPSS 14.0 statistical software. All data are presented as mean ± SD. Growth curve results were analyzed by two-way analysis of variance (ANOVA) followed by Tukey’s multiple comparison tests. Other results were analyzed by independent sample *t* test.

## Results

### Generation of stably transfected cells in which EBP50 was overexpressed or knocked down

To study the effect of EBP50 expression on EGF-stimulated cell proliferation and EGFR-mediated signal transduction pathways in breast cancer cells, we combined EBP50 gain-of-function and loss-of-function studies. Thus, MDA-MB-231 breast cancer cells, which express low levels of endogenous EBP50, were transfected with an EBP50 expression plasmid to overexpress EBP50, and MCF-7 breast cancer cells, which express high levels of endogenous EBP50, were transfected with an EBP-RNAi plasmid to knock down its expression. This was done for the purpose of observing the effect of EBP50 expression on breast cancer cells.

**Fig. 1:**
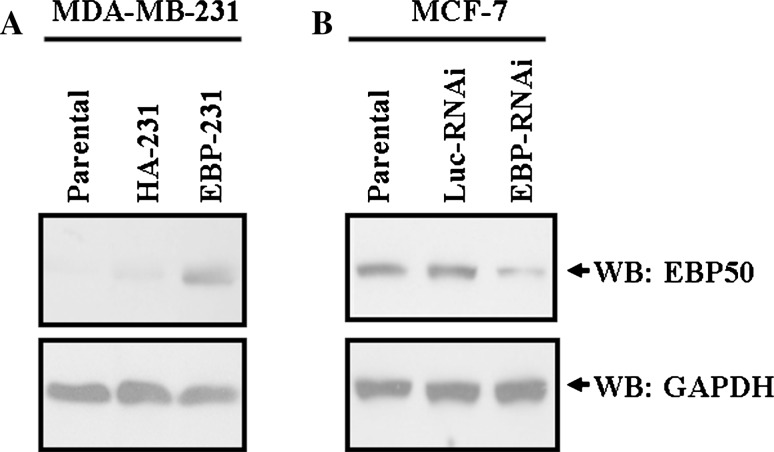
Establishment of breast cancer cells in which EBP50 expression was stably overexpressed or knocked down. **a** EBP50 was stably overexpressed in MDA-MB-231 breast cancer cells. HA-231 cells stably transfected with pBK-CMV-HA vector presented similar levels of EBP50 as that in its parental cells, and EBP50 was robustly expressed in EBP-231 cells stably transfected with pBK-CMV-HA-EBP50 constructs. **b** The expression of EBP50 was stably knocked down in MCF-7 cells. Luc-RNAi/MCF-7 cells stably transfected with Luciferase shRNA control plasmid presented the same level of EBP50 as that in parental cells, and the expression level of EBP50 in EBP-RNAi/MCF-7 cells was about 33 % as that in parental cells

The EBP50 stable transfection pool of cells, namely MDA-MB-231-HA-EBP50 (EBP-231) or its control MDA-MB-231-HA (HA-231), were generated by transfection with the neo-pBK-CMV-HA-EBP50 or neo-pBK-CMV-HA vector, respectively. Protein expression in these stable cells was verified by western blot analysis as shown in Fig. 1a. In HA-231 cells, transfection of the control vector had no effect on EBP50 expression, and similar levels of EBP50 were expressed in the parental cells. HA-tagged EBP50 protein expression was not detected in control cells (data not shown). In EBP-231 cells, exogenous HA-tagged EBP50 was robustly overexpressed.

The EBP50 stable knockdown cell line (EBP-RNAi) and its control cell line (Luc-RNAi) were generated by transfection with the pSuper.puro EBP50 RNAi plasmid or the control pSuper.puro luciferase RNAi plasmid, respectively. Verification of protein knockdown was determined by western blot analysis as shown in Fig. 1b. In Luc-RNAi cells, EBP50 expression was unaffected and EBP50 expression level was the same as that in parental cells. In EBP-RNAi cells, EBP50 expression was stably knocked down by 67 % compared to its parental cells.

### EBP50 expression suppressed EGF-induced breast cancer cell proliferation

**Fig. 2:**
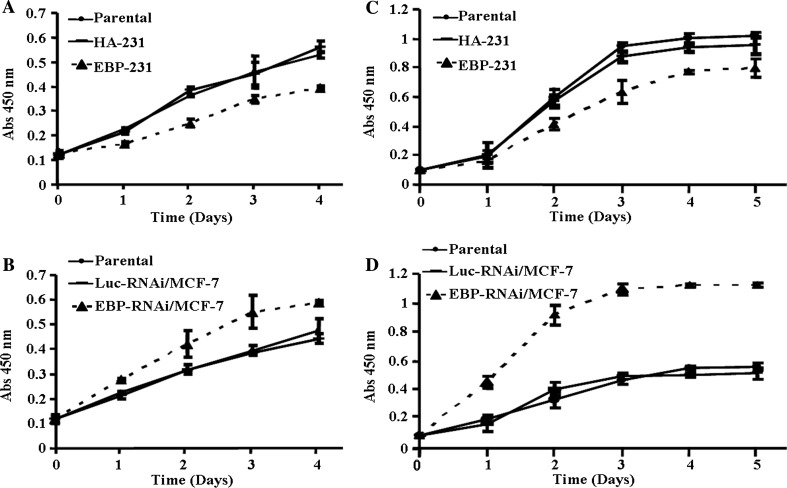
EBP50 expression suppressed EGF-induced breast cancer cell growth. **a** That EBP50 overexpression inhibited the EGF-induced proliferative response of MDA-MB-231 cells was assayed by CCK-8 method. Growth rate of EBP-231 cells was consistently slower than that of the control cells (*P* < 0.05). **b** That EBP50 knockdown enhanced EGF-induced cell proliferation of MCF-7 cells was assayed by CCK-8 method. Growth rate of EBP-RNAi/MCF-7 cells was consistently faster than that of the control cells (*P* < 0.05). **c** That EBP50 overexpression inhibited the EGF-induced proliferative response of MDA-MB-231 cells was assayed by BrdU incorporation method. DNA synthesis rate of EBP-231 cells was consistently lower than that of its control cells (*P* < 0.05). **d** That EBP50 knockdown enhanced EGF-induced cell proliferation of MCF-7 cells was assayed by BrdU incorporation method. DNA synthesis rate of EBP-RNAi/MCF-7 cells was consistently higher than that of its control cells (*P* < 0.01). All data shown are the mean ± SD of a representative experiment performed in quadruplicate (*n* = 4)

First, we detected the effect of EBP50 overexpression on EGF-induced proliferation of MDA-MB-231 cells using a CCK-8 kit to measure the number of viable cells at different time points (Fig. 2a). The results showed that overexpression of EBP50 significantly inhibited EGF-induced cell proliferation (*P* < 0.05). Compared to its vector control cells, cell proliferation was suppressed by 26 % in EBP-231 cells at day 4. This suggested that restoring EBP50 expression in EBP50 deficient MDA-MB-231 cells inhibited EGF-induced breast cancer cell proliferation. We then determined the effect of EBP50 knockdown on EGF-induced cell proliferation in MCF-7 cells. We found that EBP50 knockdown enhanced EGF-induced MCF-7 cell proliferation compared to its control cells, promoting up to 40 % increase in cell proliferation at day 4 (*P* < 0.05) (Fig. 2b). The cell counting assay confirmed this result (data not shown). Taken together, these results suggested that EBP50 inhibited EGF-induced cell proliferation in breast cancer cells.

To assess these results, cell growth curves over a longer period of time were performed with the cell culture media in the presence of serum and further stimulated with EGF using BrdU incorporation assay. As shown in Fig. 2c, d, compared to its control cells, BrdU incorporation in EBP-231 cells was significantly decreased (*P* < 0.05; Fig. 2c) and suppressed about 20 % after day 4, indicating that EBP50 expression could decrease MDA-MB-231 cell proliferation. Meanwhile, the proliferation rate of MCF-7 cell increased significantly after knockdown of EBP50 (*P* < 0.01; Fig. 2d). In the presence of serum (Fig. 2c, d), the cell growth stimulated with EGF was faster than that in serum-free medium (Fig. 2a, b), and the cell growth started to decelerate at day 3 and reached a plateau phase at day 4–5, indicating that EBP50 could really inhibit the EGF-induced cell proliferation in human breast cancer cell. Thus, both the EBP50 gain-of-function and loss-of-function studies by CCK-8 and BrdU incorporation assay demonstrated that EBP50 inhibited EGF-induced cell proliferation in MCF-7 and MDA-MB-231 breast cancer cells.

### EBP50 expression inhibited EGF-stimulated ERK1/2 and AKT phosphorylation in breast cancer cells

**Fig. 3:**
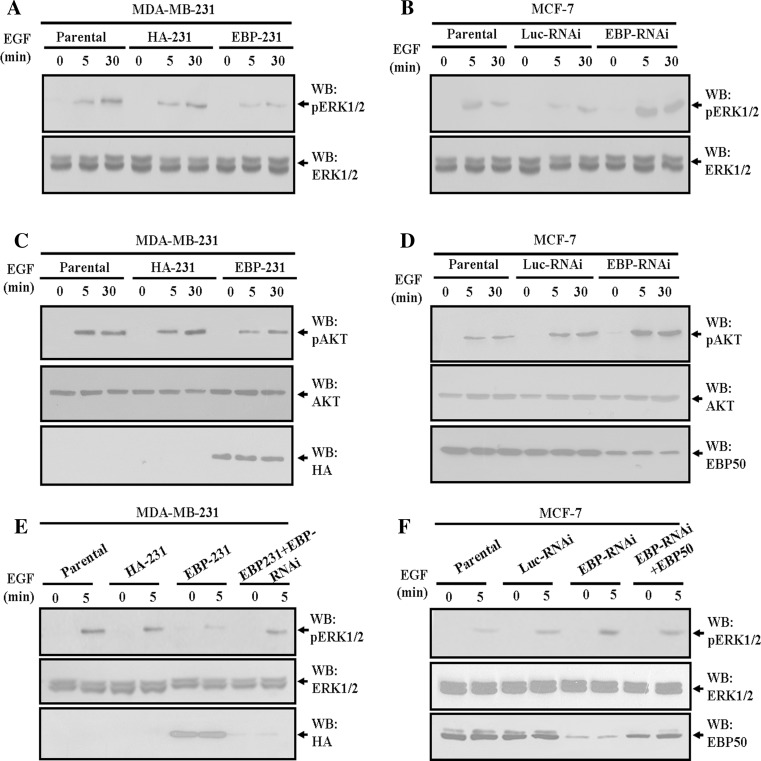
EBP50 expression inhibited EGF-stimulated ERK1/2 and AKT phosphorylation in breast cancer cells. **a** EBP50 overexpression inhibited EGF-stimulated ERK1/2 phosphorylation in MDA-MB-231 cells. ERK1/2 activation in EBP-231 cells was significantly suppressed upon 5-min or 30-min EGF treatment. **b** EBP50 knockdown enhanced EGF-stimulated ERK1/2 phosphorylation in MCF-7 cells. ERK1/2 activation in EBP-RNAi cells was significantly enhanced compared with that in its control cells upon EGF treatment. **c** EBP50 overexpression inhibited EGF-stimulated AKT phosphorylation in MDA-MB-231 cells. Phosphorylation of AKT in EBP-231 cells was significantly suppressed compared with that in its control cells upon 5-min or 30-min EGF treatment. **d** EBP50 knockdown enhanced EGF-stimulated AKT phosphorylation in MCF-7 cells. Phosphorylation of AKT in EBP-RNAi cells was significantly enhanced upon EGF treatment. **e** Knockdown of EBP50 in EBP-231 cells abrogated the inhibitory effect of EBP50 on EGF-stimulated ERK1/2 phosphorylation. When EBP50 expression in EBP-231 cells was knocked down by EBP50 siRNA, the ERK1/2 activation was increased back to similar levels to that in HA-231 cells upon 5-min EGF treatment. **f** Restoration of EBP50 expression in EBP50-knockdown cells rescued the inhibition of EBP50 on EGF-stimulated ERK1/2 phosphorylation. When EBP50 expression level in EBP-RNAi/MCF-7 cells was restored by transfection of EBP50 constructs, the ERK1/2 activation in EBP-RNAi/MCF-7 cells was decreased back to similar levels to that in Luc-RNAi/MCF-7 cells upon 5-min EGF treatment. The data presented are representative of a minimum of three independent experiments

EGF-induced increase of cell proliferation was mediated through EGFR activation. ERK1/2 mitogen-activated protein kinase (MAPK) and AKT are signaling molecules on major downstream pathways initiated by the activation of EGFR and related with cell proliferation (Lim and Cha 2011; Kim and Lim 2011). Therefore, we first explored whether EBP50 expression could inhibit ERK1/2 phosphorylation in breast cancer cells. As shown in Fig. 3a, ERK1/2 phosphorylation was inhibited in EBP-231 cells. After 5 min of EGF stimulation, ERK1/2 phosphorylation increased 12.3-fold over basal levels in the parental cells, but only 5.3-fold in EBP-231 cells. After 30 min of EGF stimulation, ERK1/2 phosphorylation increased 22.8-fold over basal levels in its parental cells, but only 9.7-fold in EBP-231 cells. Conversely, EBP50 knockdown led to enhanced EGF-stimulated ERK1/2 phosphorylation in MCF-7 cells (Fig. 3b). After 5 min of EGF stimulation, ERK1/2 phosphorylation increased 6.1-fold over basal levels in its parental cells, but more than 11.0-fold in EBP-RNAi cells. After 30 min of EGF stimulation, ERK1/2 phosphorylation increased 5.0-fold over basal levels in its parental cells, but more than 10.0-fold in EBP-RNAi cells. These results indicated that EBP50 could inhibit EGF-stimulated ERK1/2 phosphorylation in breast cancer cells.

Then, we also explored whether EBP50 expression could inhibit EGF-induced AKT phosphorylation in breast cancer cells. As shown in Fig. 3c, AKT phosphorylation was inhibited in EBP-231 cells. After 5 min of EGF stimulation, AKT phosphorylation increased 14-fold over basal levels in the HA-231 cells, but only 10-fold in EBP-231 cells. After 30 min of EGF stimulation, AKT phosphorylation increased 18-fold over basal levels in HA-231 cells, but only 13-fold in EBP-231 cells. Conversely, EBP50 knockdown led to enhanced EGF-stimulated AKT phosphorylation in MCF-7 cells. After 5 min of EGF stimulation, AKT phosphorylation increased 7.0-fold over basal levels in its parental cells, but 13-fold in EBP-RNAi cells. After 30 min of EGF stimulation, AKT phosphorylation increased 6.6-fold over basal levels in its parental cells, but more than 12-fold in EBP-RNAi cells (Fig. 3d). Overexpression of EBP50 suppressed AKT phosphorylation, and knockdown of EBP50 enhanced AKT phosphorylation, which was consistent with the results that EBP50 could inhibit ERK1/2 phosphorylation mediated by EGFR activation. Taken together, these results indicated EBP50 could inhibit EGFR-mediated downstream signaling activation.

To verify that the EGF-induced ERK1/2 phosphorylation was regulated by EBP50 expression, we further carried out rescue experiments of both knocking down EBP50 in EBP-231 cell and overexpressing EBP50 in EBP-RNAi/MCF-7 cell. The results showed that ERK1/2 was activated up to 12- and 5-fold over basal level, respectively, in HA-231 and EBP-231 cells. When EBP50 siRNA was used to knock down expression of EBP50 in EBP-231 cells, its EBP50 expression was knocked down by more than 90 % and its ERK1/2 activation was back to 10-fold over basal level as we expected, which was similar to that in HA-231 cells (Fig. 3e). Meanwhile, ERK1/2 was activated more than 11.0-fold over EBP-RNAi/MCF-7 cells, and by rescue of EBP50 expression in EBP-RNAi/MCF-7 cells, ERK1/2 phosphorylation was inhibited and returned to 7-fold over basal level, with its level similar to that in Luc-RNAi/MCF-7 cells (Fig. 3f). Taken together, these results demonstrated that EGF-stimulated downstream molecules activation was indeed regulated by EBP50 expression.

### EBP50 expression blocked EGF-induced EGFR phosphorylation in breast cancer cells

**Fig. 4:**
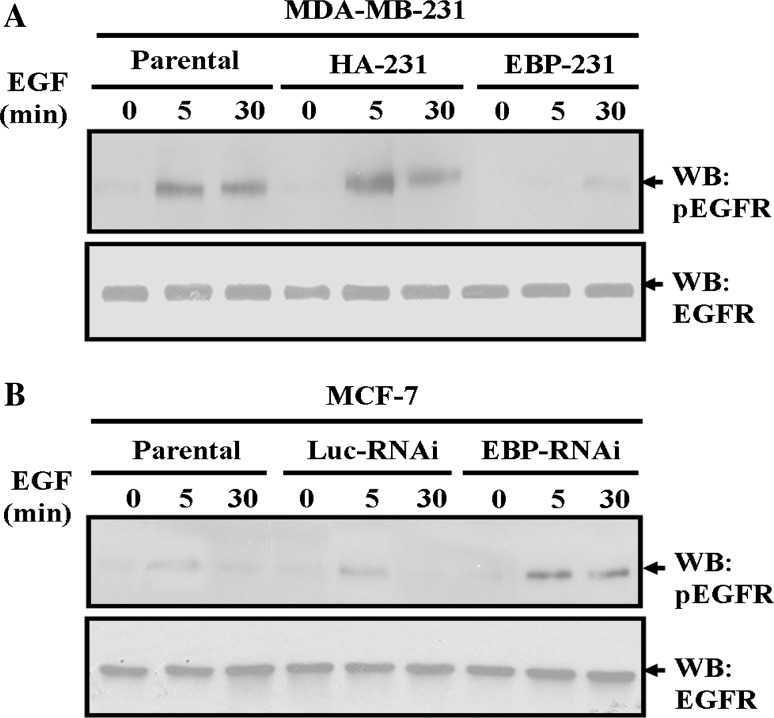
EBP50 expression blocked EGF-stimulated EGFR phosphorylation in breast cancer cells. **a** EBP50 overexpression retarded EGF-stimulated EGFR phosphorylation in MDA-MB-231 cells. EGF (100 ng/ml for 5 or 30 min)-stimulated EGFR phosphorylation in EBP-231 cells was significantly lower than that in its control cells. **b** EBP50 knockdown promoted EGF-stimulated EGFR phosphorylation in MCF-7 cells. EGF (100 ng/ml for 5 or 30 min)-stimulated EGFR phosphorylation in EBP-RNAi/MCF-7 cells was significantly higher than that in its control cells. Total expression levels of EGFR were unaffected by the different expression levels of EBP50 during EGF stimulation. The data presented are representative of a minimum of three independent experiments

EGF-stimulated ERK1/2 and AKT phosphorylation is mediated by triggering EGFR activation (Hsu et al. 2011). EBP50 was reported to interact with EGFR via its carboxyl terminal regulatory domain, which is adjacent to the autophosphorylation sites of the receptor. Therefore, it is possible that the association of EBP50 and EGFR masks the phosphorylation site of EGFR, thereby preventing EGFR activation and EGF-induced cell proliferation. To test this hypothesis, we detected the effect of EBP50 expression on EGF-stimulated EGFR phosphorylation. The overexpression of EBP50 in MDA-MB-231 cells inhibited EGF-stimulated EGFR phosphorylation (Fig. 4a). After 5 min of EGF stimulation, EGFR phosphorylation was more than 9.3-fold over basal levels in parental cells, but only less than 2.5-fold over basal level in EBP-231 cells.

The knockdown of EBP50 expression in MCF-7 cells led to induction of EGF-stimulated EGFR phosphorylation (Fig. 4b). After 5 min of EGF stimulation, EGFR phosphorylation was 3.7-fold over basal levels in parental cells, but 9.7-fold over basal levels in EBP-RNAi cells. Restoration of EBP50 expression in EBP50-knockdown MCF-7 cells reversed EGFR phosphorylation to normal levels (data not shown). Meanwhile, total expression levels of EGFR were unaffected during EGF stimulation (Fig. 4). Taken together, these results reveal that EBP50 can block EGF-induced EGFR phosphorylation in breast cancer cells.

## Discussion

In the present study, we examined the effect of EBP50 overexpression and knockdown on EGF-induced breast cancer cell proliferation. We found that overexpression of EBP50 inhibited EGF-induced breast cancer MDA-MB-231 cell proliferation, whereas knockdown of EBP50 enhanced EGF-induced breast cancer MCF-7 cell proliferation (Fig. 2). Correspondingly, EBP50 overexpression inhibited EGF-induced EGFR and downstream molecules (ERK1/2 and AKT) phosphorylation, and EBP50 knockdown enhanced EGF-induced EGFR and downstream molecules (ERK1/2 and AKT) phosphorylation (Figs. 3, 4). The rescue experiments of both knocking down EBP50 in MDA-MB-231 cells and overexpressing EBP50 in MCF-7 cells showed that the levels of pERK1/2 were rescued compared to the HA-231 and Luc-RNAi cells, respectively.

Previous studies have demonstrated that PDZ proteins regulate multiple aspects of their interacting proteins, especially their post-translational modifications. The PDZ domain protein CFTR-associated ligand (CAL) interacts with mGluR5a and regulates mGluR5a protein expression by inhibiting mGluR5a ubiquitination, thus blocking ubiquitination-dependent receptor degradation (Cheng et al. 2010). Many PDZ proteins can also influence the phosphorylation of their binding molecules. For example, PDZ protein microtubule-associated serine/threonine kinases (MASTs) can bind to phosphatase and tensin homolog deleted on chromosome 10 (PTEN) via its PDZ domain to modulate the phosphorylation of PTEN (Valiente et al. 2005). The PDZ protein PSD95 can bind to ErbB4 to regulate the phosphorylation of ErbB4 (Fujikawa et al. 2007). Chun et al. (2002) reported that the PDZ protein Na^+^/H^+^ exchanger regulatory factor 2 (NHERF2) associates with serum- and glucocorticoid-induced protein kinase 1 (SGK1) and regulates the phosphorylation of SGK1 by 3-phosphoinositide-dependent protein kinase 1 (PDK1). In this report, we also found that the PDZ protein, EBP50, regulated EGFR phosphorylation. EGFR phosphorylation is upstream of ERK1/2 and AKT activation. It was reported that pretreatment with the EGFR-specific phosphorylation inhibitor, AG1478, blocks ERK1/2 phosphorylation and decreases EGF-induced gastric mucosal cell proliferation (Osaki et al. 2011). Consistently in this study, we noticed that when MDA-MB-231 and MCF-7 breast cancer cells were pretreated with AG1478, EGF-induced EGFR phosphorylation was blocked, and resulted in the inhibition of EGF-stimulated ERK1/2 phosphorylation and cell proliferation (data not shown). We also observed that EBP50 could block EGF-induced EGFR phosphorylation, inhibit EGF-stimulated ERK1/2 phosphorylation and retard cell proliferation as well. EGFR is the only receptor through which EGF elicits ERK1/2 activation (Chen et al. 1998; DiCamillo et al. 2002), and EBP50 is reported to associate with EGFR, with the binding sites of EGFR adjacent to the receptor phosphorylation sites (Lazar et al. 2004). Thus, we speculated that EBP50 might mask the EGFR phosphorylation site to suppress EGFR downstream signaling activation, thereby hinder EGF-induced proliferation of breast cancer cells. These results provide more evidence for EBP50 as a tumor suppressor in breast cancer cell lines, and reveal an attractive mechanism that explains EBP50 tumor suppressor activity in mammary glands by counteracting the EGFR pro-oncogenic pathway.

It is known that EBP50 plays diverse roles in different tumor types and cell lines. In hepatocellular carcinoma, EBP50 promotes the development of liver cancer (Shibata et al. 2003). In biliary epithelial cells, the estrogen-inducible EBP50 protein contributes to cell proliferation (Fouassier et al. 2009). However, in mouse embryonic fibroblasts (MEF), EBP50 exerts tumor suppressor functions (Kreimann et al. 2007; Curto et al. 2007; Takahashi et al. 2006). In breast cancer, EBP50 has also been proposed to function as a tumor suppressor protein (Dai et al. 2004; Pan et al. 2006, 2008; Wheeler et al. 2011; Zheng et al. 2010b). The knockdown of EBP50 increases cell proliferation in various breast cancer cell lines and blocks cell cycle progression (Pan et al. 2006, 2008). Overexpression of EBP50 promotes cell apoptosis and inhibits serum-induced ERK1/2 activity (Zheng et al. 2010b). A recent study demonstrated that EBP50 can inhibit Wnt-dependent breast cancer cell proliferation (Wheeler et al. 2011). These diverse roles of EBP50 result from its complicated mechanism of action. The function of EBP50 is not only dependent on its binding molecules but also its subcellular localization. As an adaptor protein, EBP50 can interact with multiple types of proteins to exert tumor-promoting or tumor-suppressing functions by influencing multiple signaling pathways, including Wnt, AKT, platelet-derived growth factor receptor (PDGFR), EGFR, and ERK1/2. In hepatocellular carcinoma, EBP50 can work cooperatively with β-catenin in the nucleus to enhance Wnt signaling (Shibata et al. 2003), thus it promotes cell proliferation. In MEFs, EBP50 can stabilize β-catenin with E-cadherin in the cell membrane to suppress Wnt signaling (Kreimann et al. 2007), assist in recruiting PTEN to the cell membrane to attenuate phosphoinositide-3′-OH kinase (PI3K) activity (Takahashi et al. 2006), or bind with EGFR and neurofibromatosis 2 (NF2) at the adherens junctions to prevent EGFR from signaling (Curto et al. 2007), further to exert growth suppressing function. In breast tumors, EBP50 directly interacts with a subset of Frizzled (Fzd) receptors to inhibit canonical Wnt signaling and play tumor suppressor role (Wheeler et al. 2011). Thus, it seems that in different tissues, EBP50 binds with different binding molecules and is involved in diverse signaling pathways. However, the molecular mechanism controlling which molecule binds to EBP50 in different tissues or cell types has not been elucidated yet now.

Regulation of EGFR activity via its association with EBP50 provides cells with an additional level of control over potent mitogenic and proliferative effects induced by EGF. The results from our study are significant given the fact that EGFR is a point of convergence for several different classes of receptors (Musgrove 2004), and given the important roles that EGFR may play in triple-negative breast tumors, resistance to endocrine therapies for breast cancer, maintenance of stem-like breast tumor cells, and bone metastasis of breast cancer (Foley et al. 2010). In addition, in clinical samples, EGFR mutations [for example, T790M (Barton et al. 2010), L858R (Suzuki et al. 2007), L861Q (Yang et al. 2006)] have been detected. EGFR single nucleotide polymorphisms [SNPs: R962G, R977C, H988P (Choura et al. 2010)] have also been identified, and these mutations or SNPs are close to the EBP50 interacting site (1,037–1,065). Whether these mutations or SNPs will change its binding with EBP50 and further influence regulation of the EGFR pathway via EBP50 remains unknown. Thus, further investigation into this issue is needed.

In summary, this study demonstrated that EBP50 expression could inhibit EGF-induced breast cancer cell proliferation by blocking EGFR phosphorylation and its downstream signaling. We speculated that EBP50 masked the EGFR phosphorylation site via steric hindrance, resulting in the retardation of EGFR downstream signaling activation, thus suppressed EGF-induced proliferation of breast cancer cells. These results provide further insights into the molecular mechanism by which EBP50 regulates the development and progression of breast cancer.

## Abbreviations

EBP50: Ezrin-radixin-moesin-binding phosphoprotein-50
EGFR: Epidermal growth factor receptor
CCK-8: Cell Counting Kit-8
BrdU: 5-Bromo-2’-deoxyuridine
PDZ: Postsynaptic density-95/Discs Large/ZO-1
PTEN: Phosphatase and tensin homologue deleted on chromosome 10
ERK1/2: Extracellular signal-regulated kinase 1/2
EGF: Epidermal growth factor
SNP: Single nucleotide polymorphism
